# Lifting the Impenetrable Veil: From Yellow Fever to Ebola Hemorrhagic Fever and SARS

**DOI:** 10.3201/eid2003.131889

**Published:** 2014-03

**Authors:** Sharon Bloom

**Affiliations:** Affiliation: Centers for Disease Control and Prevention, Atlanta, Georgia, USA

Charlie Calisher is a great storyteller, and he probably has more stories to tell than anyone who has worked in the field of arbovirology. Throughout his 30-year distinguished virology career beginning at the Centers for Disease Control and Prevention to his current role as professor emeritus at the Colorado State University, Calisher has been a prolific researcher, writer, teacher, and mentor. The title phrase “lifting the impenetrable veil” originally appeared in a letter written in 1900 by Dr. Walter Reed to his wife, when he realized that he and his colleagues had shown that yellow fever was caused by a virus that was transmitted by mosquitoes. In his book, Calisher has recounted his version of arbovirus history and discovery in his unique blend of academic rigor and humorous personal interpretation.

Established as a field in the late 1950s, arbovirology is the study of arboviruses (arthropod-borne viruses). Lifting the Impenetrable Veil is organized into an introduction, 16 chapters, and an appendix that provides 25 biographic profiles of arbovirology researchers. Throughout the book the reader catches glimpses of the many colorful characters who defined arbovirology, some in shaded insets that Calisher uses to provide humorous anecdotes, usually involving himself.

I enjoyed the stories behind the many viral discoveries, especially the many lessons learned from the expansion of Venezuelan equine encephalitis virus into the Americas, the importation of West Nile Virus in the United States, and dengue reemergence worldwide. I appreciated learning about the pivotal role of the Rockefeller Foundation, founded in 1912, in professionalizing international health. I valued the clear explanations about the development of various laboratory procedures and the role they played in discovering and characterizing new viruses identified from the early 1900s to 2012. I even valued reading about the early development and current state of viral taxonomy. Most of all, I appreciated learning about fundamental epidemiologic and ecologic observations that helped elucidate aspects of arbovirus disease transmission, such as virus overwintering, transovarial transmission, and virus transmission by migrating birds. The later chapters read somewhat like a parade of researchers; however, it was still intriguing to read about these public health professionals and their pursuit of viral hemorrhagic fevers, bat-borne viruses, and Schmallenberg virus.

I once had the good fortune of meeting Dr. Calisher and feel that reading his book is not unlike listening to him in person; i.e., you can’t help but smile and learn from his informative, opinionated, yet always down-to-earth storytelling. The experience is eclectic and memorable. Be warned: this book has minor shortcomings that are typical of self-published books, such as uneven editing. However, I strongly recommend this book for anyone interested in the history of virology, particularly researchers in the field of arbovirology.

