# Correction: Vol. 20, No. 3

**DOI:** 10.3201/eid2011.C22011

**Published:** 2014-11

**Keywords:** errata, erratum, correction

In the review of Lifting the Impenetrable Veil: From Yellow Fever to Ebola Hemorrhagic Fever and SARS (S. Bloom), the affiliation for Charlie Calisher was misstated. He is professor emeritus at Colorado State University. The article has been corrected online (http://wwwnc.cdc.gov/eid/article/20/3/13-1889.htm).

